# Giant Coronary Artery Aneurysms

**DOI:** 10.7759/cureus.15611

**Published:** 2021-06-12

**Authors:** Megan C Smith, Alex Schneller, Aniruddha Singh, Rahil Rafeedheen

**Affiliations:** 1 Cardiology, University of Kentucky, Bowling Green, USA; 2 Cardiology, University of Kentucky School of Medicine, Bowling Green, USA; 3 Interventional Cardiology, The Medical Center, Bowling Green, USA

**Keywords:** coronary artery bypass grafting (cabg), coronary artery aneurysm, abdominal aortic aneurysm, popliteal artery aneurysm, multivessel coronary artery disease (mvcad)

## Abstract

We present the case of a patient with giant coronary artery aneurysm. He has underlying severe coronary atherosclerosis and concomitant aneurysms of the abdominal aorta and popliteal artery. Our patient was treated surgically in the past due to underlying severe atherosclerosis. Despite bypass, his coronary aneurysms continued to enlarge. There is a lack of randomized trials regarding management to guide the decision-making process. Our case describes the work-up and treatment of a patient with giant coronary artery aneurysm requiring urgent orthopedic surgery.

## Introduction

Coronary artery aneurysms (CAAs) are scarce among the cardiovascular diseases. Giant CAAs are even more rare. The prevalence of CAA is reported to be 1.4% and giant CAA even lower at 0.02% [1]. The most common cause of CAA is atherosclerosis (52%), followed by congenital (17%), inflammatory disorders (17%), infectious (11%), connective tissue disorders (<10%), drug-related, trauma, and iatrogenic [2-3]. Most patients remain asymptomatic. However, the most common clinical manifestation is stable angina [1,4]. To constitute an aneurysm, the vessel lumen must be greater than 50% of its normal width in a localized, irreversible fashion. For giant CAAs, the lumen must be four times greater or greater than 8 mm [1,3]. Coronary angiography has been the diagnostic modality of choice; however, newer modalities, such as coronary magnetic resonance angiogram and coronary computed tomography angiogram, are becoming more readily available and ever useful choice for follow-up [3].

## Case presentation

**Figure 1:**
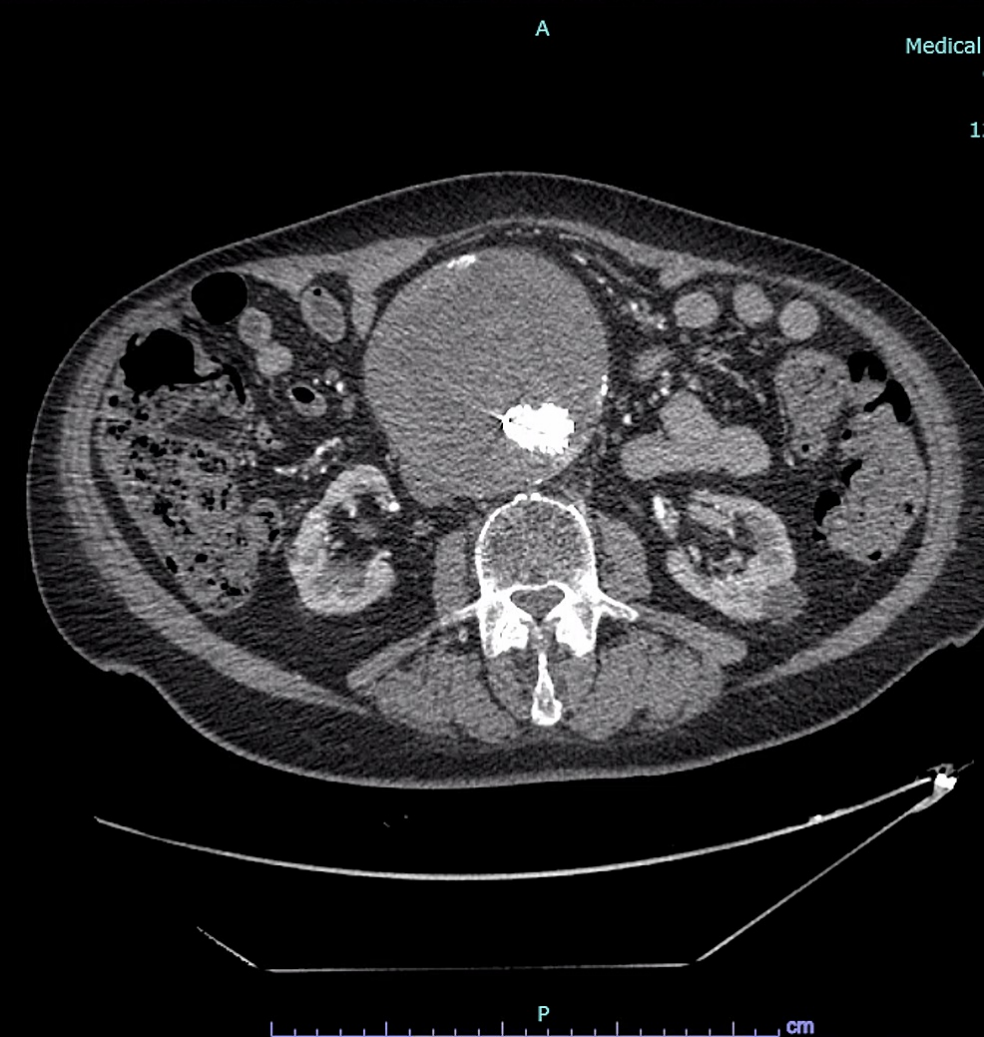
Computed tomography angiography of the abdomen and pelvis revealing large abdominal aortic aneurysm with endograft in place

An 81-year-old Caucasian man presented to our hospital after a mechanical fall resulting in a right femur periprosthetic fracture. His past medical history was pertinent for coronary artery bypass grafting (CABG) and aortic valve replacement with a 27-mm Saint Jude Biocor bioprosthetic valve due to severe aortic insufficiency in 2007, paroxysmal atrial fibrillation, hypertension, hyperlipidemia, diabetes mellitus, former tobacco abuse, right popliteal aneurysm, and abdominal aortic aneurysm measuring up to 12 cm x 12 cm status post-endovascular aortic repair in 2011 (Figure 1).

**Figure 2:**
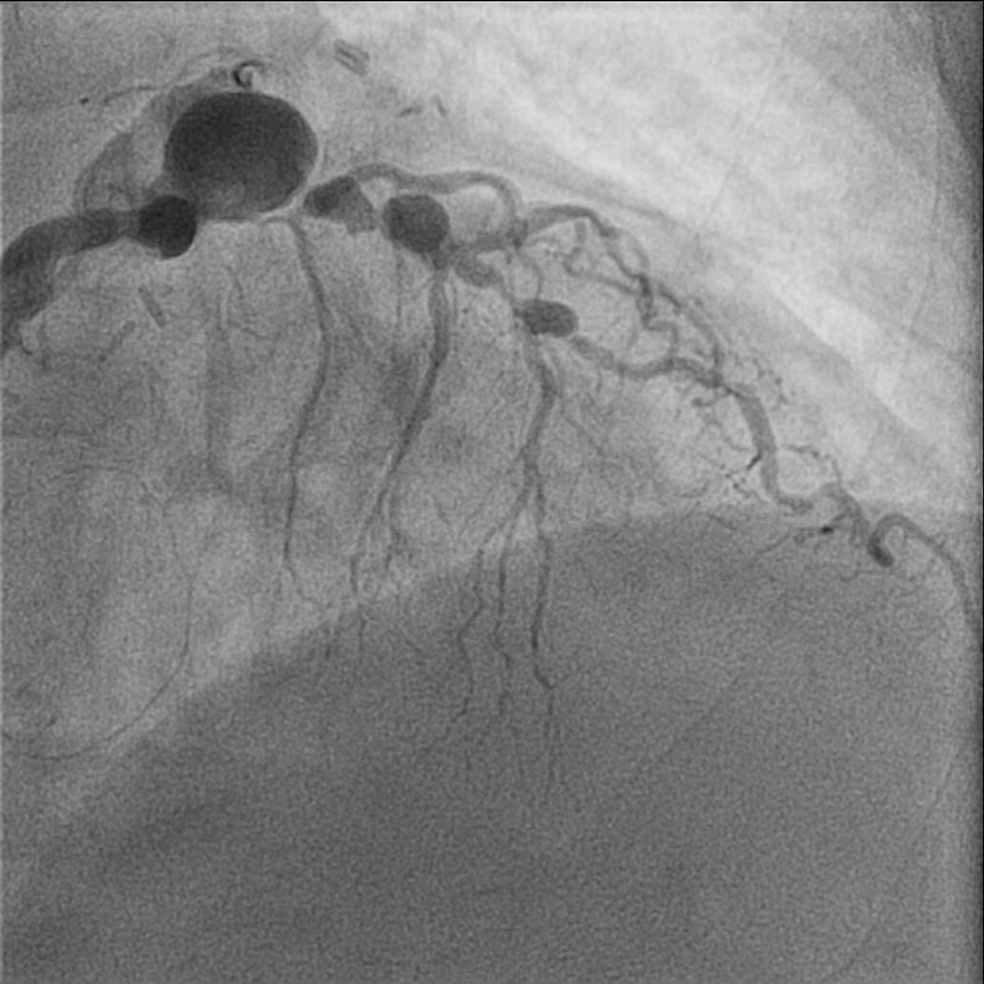
Coronary angiography highlighting aneurysms of the left anterior descending artery in right anterior oblique caudal projection

He reported intermittent chest pressure and dyspnea with mild exertion. He can walk about half of a block before stopping to rest due to dyspnea. Transthoracic echocardiogram showed preserved ejection fraction of 60-65%, stage 1 diastolic dysfunction, well-seated bioprosthetic aortic valve with normal gradients, and no other major valvular abnormalities. A regadenason nuclear stress test was performed and it revealed a fixed inferior wall defect and significant transient ischemic dilation, prompting further investigation with coronary and bypass angiography. Coronary angiography revealed numerous aneurysmal dilations, most notably the proximal left anterior descending artery (LAD), as well as severe atherosclerosis lesions (Figure 2).

**Figure 3:**
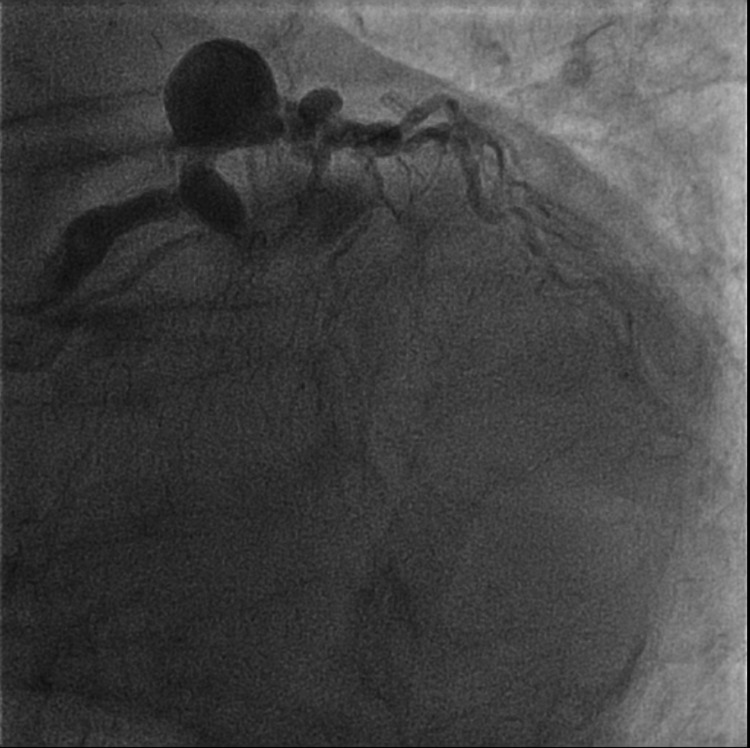
Coronary angiography revealing complete occlusion of the proximal left circumflex artery in left anterior oblique caudal projection

The left circumflex artery (LCX) was occluded proximally (Figure 3).

**Figure 4:**
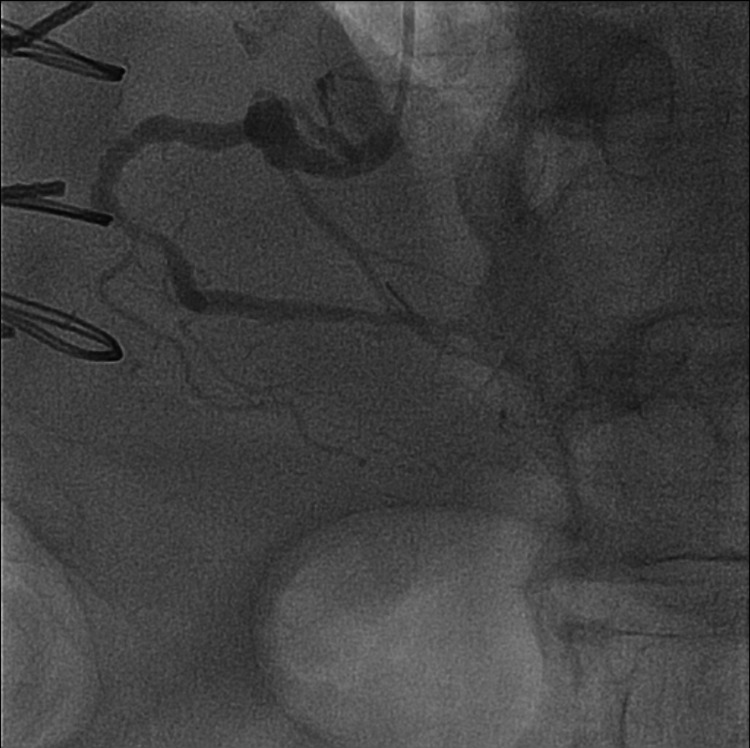
Coronary angiography of the right coronary artery in left anterior oblique cranial projection

Bypass angiography revealed patent grafts to the LAD, obtuse marginal branch, and right coronary artery (RCA) (Figure 4).

Given his age, underlying comorbidities, and patent bypass grafts, we opted to treat him medically with optimization of medical therapy, which included anti-platelet, angiotensin receptor antagonists, and statins.

He proceeded with open reduction internal fixation of the right periprosthetic femur fracture. The surgery was uncomplicated, and his post-operative course was relatively unremarkable. He was discharged to an inpatient rehabilitation facility to continue recovery.

## Discussion

CAAs most frequently affect the RCA (40.4%), followed by the LAD (32.3%), LCX (23.4%), and least commonly the left main (LM) coronary artery (3.5%) [1,2]. The most common cause of CAAs is atherosclerosis in adults and Kawasaki disease in children. Aneurysms attributed to atherosclerosis are typically multiple, involving more than one coronary artery, as compared with traumatic, congenital, or dissecting aneurysms that are typically solitary [1].

The pathogenesis is not completely understood but likely similar to that of large vessels and involves destruction of arterial media, thinning of the arterial wall, and increases in wall stress. Aneurysmal segments are characterized by elevated concentrations of proinflammatory cytokines and matrix metalloproteinases (MMPs) that are capable of degrading essentially all components of the arterial wall matrix [1,3]. These inflammatory cells can reside inside plaques, secreting cytokines, further increasing inflammation and producing proteases that help to destabilize plaque and further damage the extracellular matrix [1].

Most patients with CAAs are asymptomatic; however, the most common presenting symptom is angina. Myocardial infarction and sudden death can also occur [1,3,4]. Coronary angiography is the “gold standard” for diagnosis of CAA [1]. Other methods include echocardiography, CT, and MRI, which are increasing in popularity as non-invasive methods of follow-up for these patients [4].

Treatment options of CAAs include medical management, surgical resection or bypass, and percutaneous intervention. Management decisions are complicated by the absence of randomized trials to evaluate the different management strategies. Medical therapy includes anti-platelets and anticoagulants, angiotensin receptor antagonists, and statins with the goal of preventing thromboembolic complications and targeting MMPs. Percutaneous intervention is a relatively newer treatment option with limited data. Most commonly used methods include conventional stent placement and coil embolization. Surgical management is reserved for symptomatic patients with obstructive coronary disease and aneurysms at high risk for rupture [1,3]. High-risk features include thrombolysis in myocardial infarction (TIMI) 0 or 1 flow in the aneurysmal vessel, hemodynamic instability, sustained ventricular tachycardia, and recurrent angina, none of which our patient currently displayed [3].

Our patient’s largest CAA in the proximal LAD measured 16.7 mm x 13.6 mm. The average size of the coronary arteries are 4.12 ± 0.68 mm for the LM, 2.26 ± 0.41 mm for the LAD, 2.14 ± 0.43 mm for the LCX, and 2.95 ± 0.60 mm for the RCA [5]. He had previously undergone CABG in 2007, at which time he had severe coronary artery disease and ectasia. His coronaries have continued to enlarge since that time. Despite the significant size of his CAAs, no surgical or percutaneous intervention was recommended. All bypass grafts were patent and functioning appropriately. His symptoms were felt to be most likely due to deconditioning and likely underlying chronic lung pathology from years of tobacco abuse. Medical therapy for coronary artery disease was optimized.

## Conclusions

Giant CAAs are rare, and most CAAs are found incidentally. There are several treatment options including percutaneous, surgical, and medical management; however, randomized trials regarding management are scarce. Our case represents an initial surgical management followed by medical therapy prior to a non-cardiac surgery to add to the body of literature regarding treatment of these rarely seen aneurysms.
